# Supplementary material for: Efficient phonon cascades in WSe2 monolayers
Source: Nat Commun. 2021 Jan 22;12:538. doi: 10.1038/s41467-020-20244-7 (PMC7822848; doi:10.1038/s41467-020-20244-7)
Supplement: Supplementary file 1 — Supplementary Information [file 41467_2020_20244_MOESM1_ESM.pdf]

Efficient phonon cascades in a monolayer semiconductor

Ioannis Paradisanos[1,2,*] Gang Wang[2,3], Evgeny M. Alexeev[2], Alisson R. Cadore[2], Xavier Marie[1], Andrea C. Ferrari[2,†] Mikhail M. Glazov[4,‡] and Bernhard Urbaszek[1§]

[1] *Université de Toulouse, INSA-CNRS-UPS, LPCNO, 135 Avenue Rangueil, 31077 Toulouse, France*

[2] *Cambridge Graphene Centre, University of Cambridge, Cambridge CB3 0FA, U.K.*

[3] *Key Lab of Advanced Optoelectronic Quantum Architecture and Measurement (MOE), School of Physics, Beijing Institute of Technology, Beijing 100081, China and*

[4] *Ioffe Institute, 194021, St.-Petersburg, Russia*

Supplementary Note 1. Raman and PL spectra fitting

Supplementary Figure 1a-c reports optical microscopy images of some of the studied samples: (a) 1L-WSe_2 on Au and suspended, (b) 1L-WSe_2 on SiO_2/Si, (c) 1L-WSe_2 on hBN. Representative PL spectra collected at $T = 295$ K for 514 nm excitation are shown in Supplementary Figure 1d, showing the A-exciton resonance.

Supplementary Figure 2a plots representative data fits. The spectrum, measured at 295 K at 532 nm, is shown with black dots. Lorentzian functions are used to fit the Raman peaks (FWHM∼1-10cm^{-1}) and are plotted in blue. The residual spectral weight is also fitted with Lorentzians and results into the broader (FWHM∼50-80cm^{-1}) peaks of the hot PL, shown in red. A flat baseline is taken into account for the whole energy scale, since the background in the S spectral range increases due to the higher intensity of the cascades in S compared to AS. A fitting example is shown in Supplementary Figure 2b. The Lorentzians naturally overlap, thus creating an asymmetric broad background (indicated by yellow dashed lines in Supplementary Figure 2b).

* paradeis@insa-toulouse.fr

† acf26@eng.cam.ac.uk

‡ glazov@coherent.ioffe.ru

§ urbaszek@insa-toulouse.fr

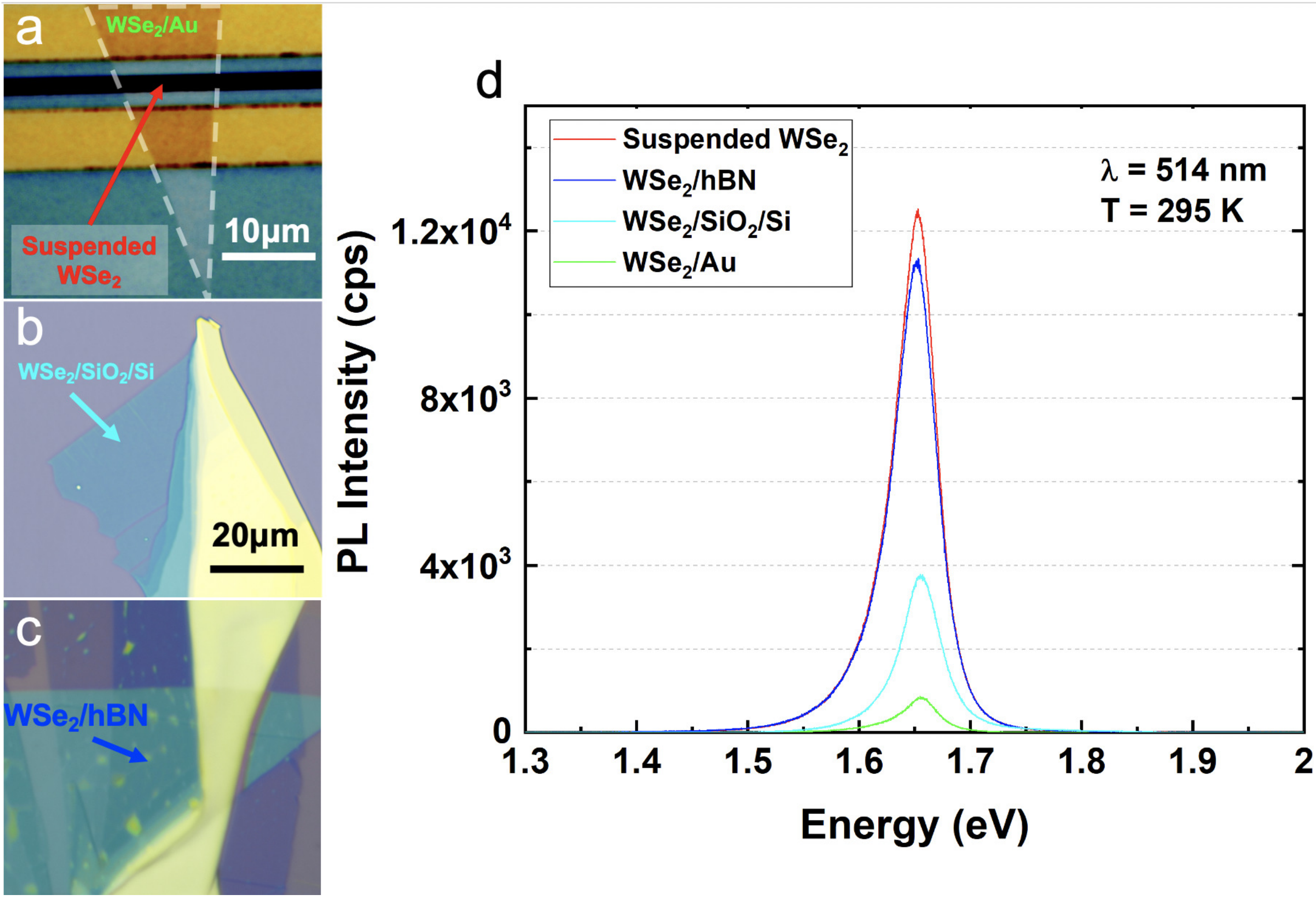

Supplementary Figure 1. Optical images of (a) 1L-WSe_2 on Au and suspended, (b) 1L-WSe_2 on SiO_2/Si, (c) 1L-WSe_2 on hBN. Representative PL spectra collected at 295K for 514 nm excitation are shown in (d). Stronger PL intensities are observed in the suspended and hBN cases, due to the suppression of substrate-related doping and/or disorder effects.

Supplementary Note 2. Diagrammatic calculation of Stokes scattering at 0K

For 0K, we calculate the S emission. The light scattering cross-section can be written as (disregarding polarization dependence)[1–3]:

$$\sigma(\omega_i, \omega_f) = s \sum_k S_k(\omega_i, \omega_f), \tag{1}$$

where s is a prefactor non-resonantly dependent on the initial and final frequencies, $S_k(\omega_i, \omega_f)$ is the effective cross-section due to the participation of k phonons in the intermediate states, shown by green arrows in Supplementary Figure 3a. We assume that photoexcitation results in the generation of excitons due to their high (hundreds of meV) binding energies[4]. The

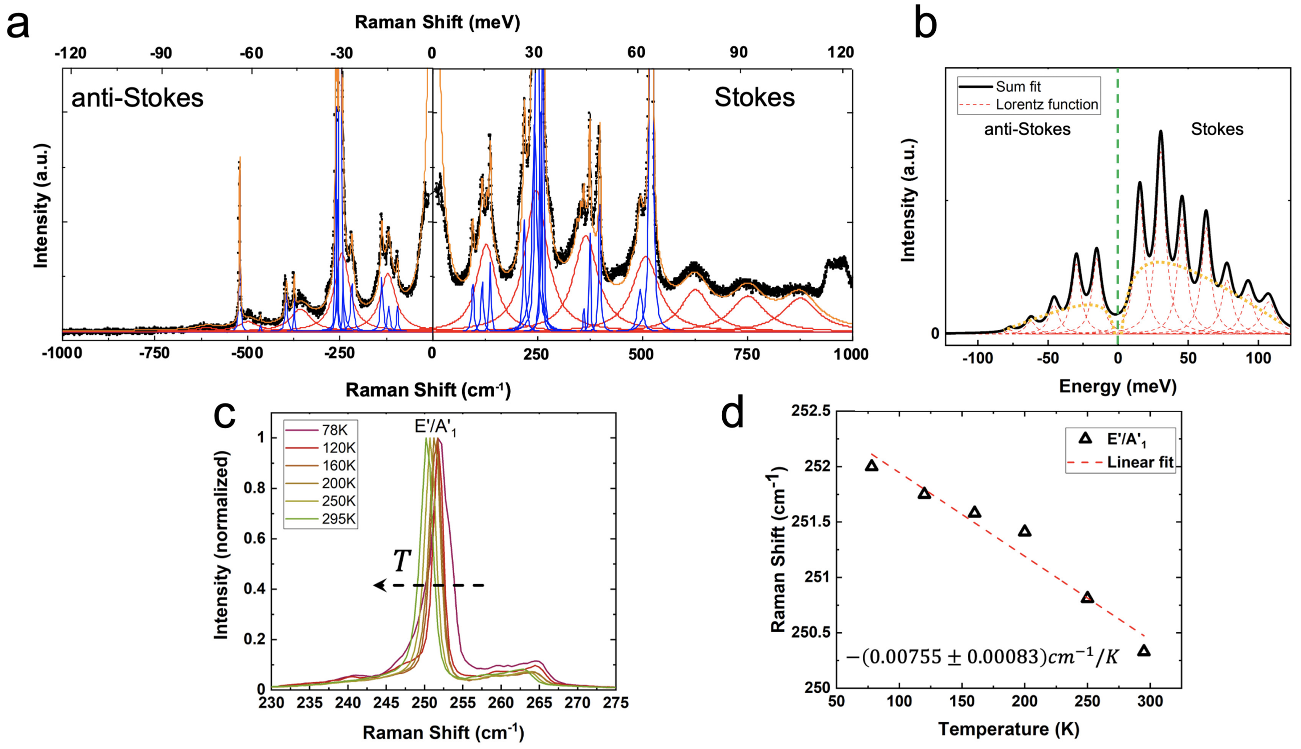

Supplementary Figure 2. (a) Fitted 1L-$WSe_2/SiO_2/Si$ spectrum, collected at 295K and 532nm. (b) Lorentzian (red lines) S/AS sum fit. Yellow dashed lines indicate the asymmetric background.(c) Normalized Raman intensity of E',A'_1 mode at different T. (d) Pos(E',A'_1) as a function of T and linear fit.

description in the case of unbound e-h pairs is similar and outlined below.

The calculation of the partial contributions $S_k(\omega_i, \omega_f)$ can be performed in the framework of the diagram technique of Refs.[1, 2, 5]. We extend this to the two-dimensional case, with the exciton-phonon interaction described by the matrix element M_0, independent of wavevector. Phonons, as in Refs.[6, 7], are considered as dispersionless. This is reasonable for 1L-TMDs, where Fröhlich coupling is suppressed[8, 9]. Following Ref.[9], we introduce the coupling constant:

$$\beta = \frac{2\mathcal{S}m|M_0|^2}{\hbar^3\Omega}, \tag{2}$$

where $\mathcal{S}$ is the normalization area, m the exciton translational mass and Ω the phonon frequency. We focus on one excitonic band (stemming from 1s excitons), and disregard the multivalley structure of 1L-TMDs[4] for simplicity. The exciton damping rate in the state

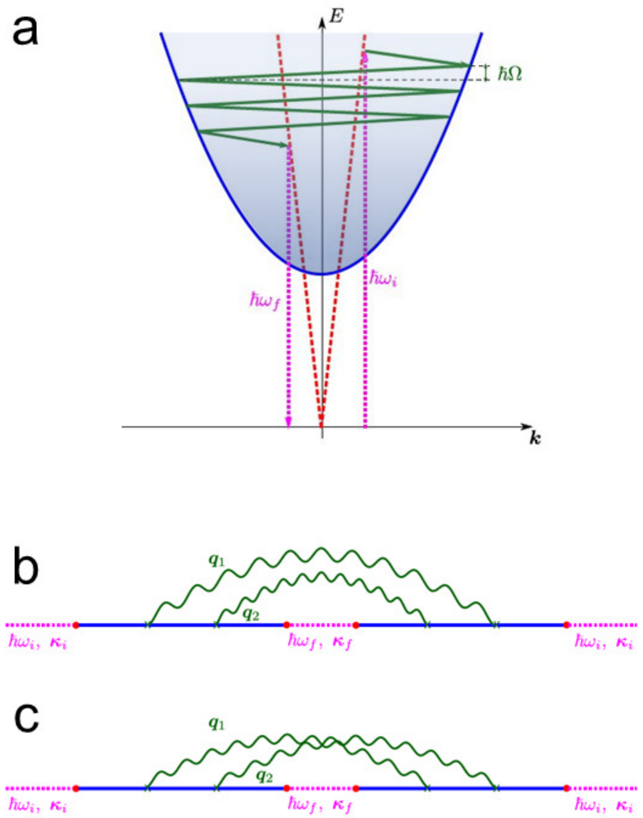

Supplementary Figure 3. (a) Scheme of multiphonon process in the resonant Raman scattering of light by excitons/PL. The incident, $\hbar\omega_i$, and outgoing, $\hbar\omega_f$, photons are shown by the dotted magenta vertical arrows. The phonons participating in the cascade are shown by the green arrows. The e-h dispersion curve is the filled blue parabola. The light cone is shown by red dashed lines. (b-c) Feynman diagrams corresponding to two-phonon processes. The dotted magenta lines are the photon Green functions in the free space. Blue lines are exciton Green functions (retarded ones at the left-hand side, before the internal photon function, and advanced at the right-hand side). Wiggly lines are phonon Green functions D^-.

with wavevector $\boldsymbol{k}$ due to emission of dispersionless phonons is given by:

$$\gamma_{o,k} = \frac{1}{2\tau_{o,k}} = \frac{\pi}{\hbar}\sum_{\boldsymbol{k'}} |M_0|^2 \delta(E_k - E_{k'} - \hbar\Omega) = \frac{\beta\Omega}{4}\Theta(E_k - \hbar\Omega), \tag{3}$$

where $E_k = \hbar^2 k^2/2m$ is the exciton dispersion, $\Theta(x)$ is the Heaviside step function. The total damping rate of the exciton $\gamma_k > \gamma_{o,k}$ contains also the contributions due the interaction with acoustic phonons[10, 11], disorder[12], non-radiative[12] and radiative (for states within

the light cone) damping[13].

Correspondingly, the exciton retarded Green functions read:

$$G(\varepsilon, \boldsymbol{k}) = \frac{1}{\varepsilon - E_k + \mathrm{i}\gamma_k}. \tag{4}$$

By labelling Γ_q the phonon damping, the Green functions become:

$$\begin{aligned} D(\omega, \boldsymbol{q}) &= \frac{1}{\hbar\omega - \hbar\Omega + \mathrm{i}\Gamma_q}, \\ D^-(\omega, \boldsymbol{q}) &= D(\omega, \boldsymbol{q}) - D^*(\omega, \boldsymbol{q}) = \frac{-2\mathrm{i}\Gamma_q}{(\hbar\omega - \hbar\Omega)^2 + \Gamma_q^2}. \end{aligned} \tag{5}$$

Supplementary Figure 3a illustrates the relevant diagrams describing the two-phonon process:

$$\begin{aligned} S_2^{(a)} = -|M_0|^4 \sum_{\boldsymbol{k}} \int_{-\infty}^{\infty} \frac{d\omega}{2\pi} G(\hbar\omega_i - E_1, \boldsymbol{\kappa}_i) G(\hbar\omega_i - E_1 - \hbar\omega, \boldsymbol{k}) G(\hbar\omega_f - E_1, \boldsymbol{\kappa}_f) \\ \times D^-(\omega, \boldsymbol{\kappa}_i - \boldsymbol{k}) D^-(\omega_i - \omega_f - \omega, \boldsymbol{k} - \boldsymbol{\kappa}_f) \\ \times G^*(\hbar\omega_i - E_1, \boldsymbol{\kappa}_i) G^*(\hbar\omega_i - E_1 - \hbar\omega, \boldsymbol{k}) G^*(\hbar\omega_f - E_1, \boldsymbol{\kappa}_f) \\ = -|M_0|^4 \left|G(\hbar\omega_i - E_1, \boldsymbol{\kappa}_i)\right|^2 \left|G(\hbar\omega_f - E_1, \boldsymbol{\kappa}_f)\right|^2 \\ \times \sum_{\boldsymbol{k}} \int_{-\infty}^{\infty} \frac{d\omega}{2\pi} D^-(\omega, \boldsymbol{\kappa}_i - \boldsymbol{k}) D^-(\omega_i - \omega_f - \omega, \boldsymbol{k} - \boldsymbol{\kappa}_f) \left|G(\hbar\omega_i - E_1 - \hbar\omega, \boldsymbol{k})\right|^2. \end{aligned} \tag{6a}$$

$$\begin{aligned} S_2^{(b)} = -|M_0|^4 \left|G(\hbar\omega_i - E_1, \boldsymbol{\kappa}_i)\right|^2 \left|G(\hbar\omega_f - E_1, \boldsymbol{\kappa}_f)\right|^2 \\ \times \sum_{\boldsymbol{k}} \int_{-\infty}^{\infty} \frac{d\omega}{2\pi} D^-(\omega, \boldsymbol{\kappa}_i - \boldsymbol{k}) D^-(\omega_i - \omega_f - \omega, \boldsymbol{k} - \boldsymbol{\kappa}_f) G(\hbar\omega_i - E_1 - \hbar\omega, \boldsymbol{k}) G^*(\hbar\omega_f - E_1 + \hbar\omega, \boldsymbol{\kappa}_i + \boldsymbol{\kappa}_f - \boldsymbol{k}). \end{aligned} \tag{6b}$$

where $E_1 = E_g - E_{b,1s}$ is the $1s$ exciton excitation energy, $E_{b,1s}$ is its binding energy.

Supplementary Figure 3a describes the process where two phonons are emitted one after another. Supplementary Figure 3b shows the quantum interference of two-photon emission processes[1]. When $\Gamma_q \gg \gamma_k$, the contribution in Supplementary Figure 3b, given by Eq.(6b), is smaller by a factor γ_k/Γ_q, compared to the non-crossing contribution in Supplementary Figure 3a.

We now focus on the more realistic case where $\Gamma_q \ll \gamma_k$. For simplicity, we disregard the k-dependence of γ_k and the q-dependence of Γ_q and omit the corresponding subscripts.

Thus:

$$S_2^{(a)} = \frac{4\Gamma}{4\Gamma^2 + (2\Omega - \omega_i + \omega_f)^2} |M_0|^2 \left|G(\hbar\omega_i - E_1, \boldsymbol{\kappa}_i)\right|^2 \left|G(\hbar\omega_f - E_1, \boldsymbol{\kappa}_f)\right|^2 \times \frac{1}{\hbar} \sum_{\boldsymbol{k}} \frac{|M_0|^2}{\hbar^2\gamma^2 + (\hbar\Omega + \hbar\omega_f - E_1 - E_k)^2} \tag{7a}$$

$$S_2^{(a)} = \frac{4\Gamma}{4\Gamma^2 + (2\Omega - \omega_i + \omega_f)^2} |M_0|^2 \left|G(\hbar\omega_i - E_1, \boldsymbol{\kappa}_i)\right|^2 \left|G(\hbar\omega_f - E_1, \boldsymbol{\kappa}_f)\right|^2 \times \frac{1}{\hbar} \sum_{\boldsymbol{k}} \frac{|M_0|^2}{(\hbar\omega_f + \hbar\Omega - E_1 - E_k + \mathrm{i}\gamma)(\hbar\omega_f + \hbar\Omega - E_1 - E_{k'} - \mathrm{i}\gamma)}, \tag{7b}$$

with $\boldsymbol{k}' = \boldsymbol{\kappa}_i + \boldsymbol{\kappa}_f - \boldsymbol{k}$. Note that

$$\frac{1}{\hbar} \sum_{\boldsymbol{k}} \frac{|M_0|^2}{\hbar^2\gamma^2 + (\hbar\Omega + \hbar\omega_f - E_1 - E_k)^2} = \frac{\gamma_o}{\gamma}, \tag{8a}$$

$$\frac{1}{\hbar} \sum_{\boldsymbol{k}} \frac{|M_0|^2}{(\hbar\omega_f + \hbar\Omega - E_1 - E_k + \mathrm{i}\gamma)(\hbar\omega_f + \hbar\Omega - E_1 - E_{k'} - \mathrm{i}\gamma)} = \frac{\gamma_o/\gamma}{\sqrt{1 + \left(\frac{|\boldsymbol{\kappa}_i + \boldsymbol{\kappa}_f| v}{2\gamma}\right)^2}}, \tag{8b}$$

where $v = \sqrt{2(\hbar\omega_f + \hbar\Omega - E_1)/m}$. Thus

$$S_2^{(a)} = \frac{4\Gamma}{4\Gamma^2 + (2\Omega - \omega_i + \omega_f)^2} |M_0|^2 \frac{\gamma_o}{\gamma} \left|G(\hbar\omega_i - E_1, \boldsymbol{\kappa}_i)\right|^2 \left|G(\hbar\omega_f - E_1, \boldsymbol{\kappa}_f)\right|^2, \tag{9a}$$

$$S_2^{(b)} = \frac{S_2^{(a)}}{\sqrt{1 + \left(\frac{|\boldsymbol{\kappa}_i + \boldsymbol{\kappa}_f| v}{2\gamma}\right)^2}}. \tag{9b}$$

We get:

$$\frac{|\boldsymbol{\kappa}_i + \boldsymbol{\kappa}_f| v}{2\gamma} \sim \frac{l_{\text{eff}}}{\lambda},$$

where l_{eff} is the effective mean free path of the exciton, and λ is the characteristic wavelength of light. In backscattering in plane, since scattered light is emitted by both 1L-WSe_2 sides:

$$\boldsymbol{\kappa}_f = -\boldsymbol{\kappa}_i, \tag{10}$$

the diagram with crossed phonon lines doubles the result stemming from the diagram in Supplementary Figure 3b. This is due to coherent backscattering (or weak localization) effect[14, 15]. Otherwise the contribution of the diagram Supplementary Figure 3b is negligible, provided that $l_{\text{eff}} \gg \lambda$. While the latter condition may not be strictly fulfilled in

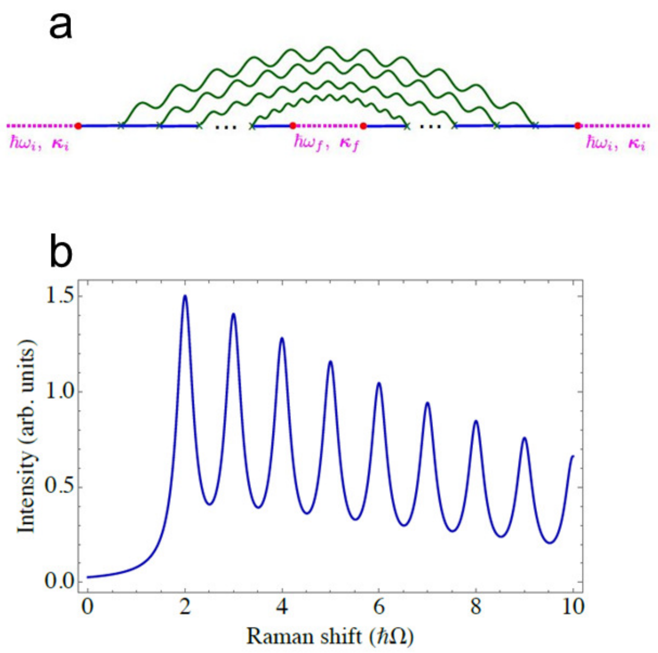

Supplementary Figure 4. (a) Multiphonon scattering in the non-crossing approximation. (b) Hot PL for $\gamma_o/\gamma = 0.9$, $\Gamma/\Omega = 0.1$, $K = 10$.

1L-TMDs[15], we disregard the contributions due to the crossed diagrams to provide an analytical model.

Importantly, the phonon scattering is resonant, taking place via real intermediate states and, accordingly, the scattering cross-section acquires a factor $\frac{\gamma_o}{\gamma}$, which gives the probability for an exciton to emit a phonon during its lifetime in a state with wavevector $\boldsymbol{k}$. If inelastic scattering is dominated by a single phonon mode, γ_o/γ can be close to unity.

We now consider multiphonon processes. Within the non-crossing approximation, we take into account the diagrams where the phonon propagators do not cross, i.e., we disregard the interference of the phonons. We can thus sum the contributions of the diagrams in Supplementary Figure 4a with $k = 2, 3, \ldots$ phonon lines.

The maximum number of phonons involved in the process is given by:

$$K = \left\lfloor \frac{\hbar\omega_i - E_1}{\hbar\Omega} \right\rfloor, \tag{11}$$

Performing the calculations analogous to those presented above we arrive at:

$$\sigma(\omega_i, \omega_f) = \sigma_0(\omega_i, \omega_f) \sum_{k=2}^{K} \frac{1}{\pi} \frac{2\Gamma}{4\Gamma^2 + (k\Omega - \omega_i + \omega_f)^2} \left(\frac{\gamma_o}{\gamma}\right)^{k-1}, \tag{12}$$

where $\sigma_0(\omega_i, \omega_f)$ is a smooth function of frequency.

for $K \to \infty$ we get:

$$\sigma(\omega_i, \omega_f) = \sigma_0(\omega_i, \omega_f)\frac{x}{\pi} \times \Re\left\{\frac{{}_2F_1\left(1, \frac{-2i\Gamma+2\Omega-\omega_i+\omega_f}{\Omega}, \frac{-2i\Gamma+3\Omega-\omega_i+\omega_f}{\Omega}, x\right)}{2\Gamma + i(2\Omega - \omega_i + \omega_f)}\right\}, \tag{13}$$

with ${}_2F_1(a, b, c, x)$ the hypergeometric function, and $x = \gamma_o/\gamma$.

A typical calculated spectrum at 0K considering the S component of the emission is in Supplementary Figure 4b. Each cascade step provides a factor γ_o/γ to the scattering cross-section. If the scattering rates γ_o and γ are energy dependent, the S component of emission at the jth step is given by the products:

$$\mathrm{I}_j \propto \prod_{k=2}^{j} \frac{\gamma_o(k)}{\gamma(k)}, \tag{14}$$

with the argument k denoting the step of the cascade (i.e., the energy) where the corresponding scattering rate is taken.

In the presence of static disorder, or quasi-elastic acoustic phonon scattering with negligible energy transfer, additional diagrams with the corresponding scattering processes should be taken into account. To illustrate that elastic scattering processes do not suppress oscillations in the Raman and hot PL, we consider exciton scattering by static impurities with scattering rate γ_{imp}, with γ, the total exciton scattering rate. Taking into account diagrams similar to Supplementary Figure 4a, but with impurity lines, γ_o/γ is renormalized by elastic scattering as:

$$\frac{\gamma_o}{\gamma} \to \frac{\gamma_o}{\gamma}\sum_{n=0}^{\infty}\left(\frac{\gamma_{\text{imp}}}{\gamma}\right)^n = \frac{\gamma_o}{\gamma - \gamma_{\text{imp}}}. \tag{15}$$

This means that for elastic scattering the exciton energy does not change, thus it does not smear-out the intensity oscillations.

Supplementary Note 3. Kinetic equation

The non-crossing approximation corresponds to a kinetic equation model where the exciton dynamics after optical excitation is described by the Boltzmann equation in the form[16]:

$$\gamma' f(\boldsymbol{k}) = \sum_{\boldsymbol{k}'} W_{\boldsymbol{k}\boldsymbol{k}'}[f_{\boldsymbol{k}'} - W_{\boldsymbol{k}'\boldsymbol{k}} f_{\boldsymbol{k}}] + g_{\boldsymbol{k}}. \tag{16}$$

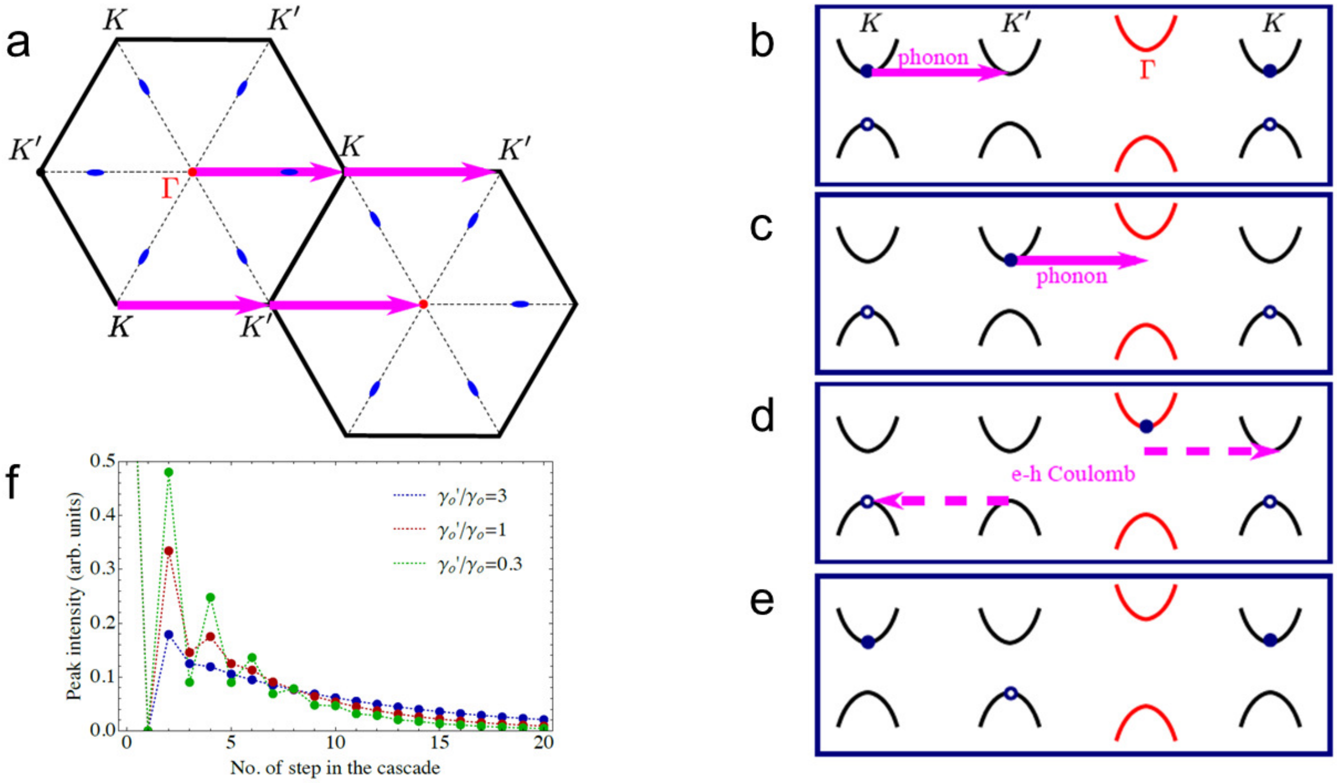

Supplementary Figure 5. Schematic band diagram and scattering pathways in e-h representation. (a) Extended BZ with K valley shown twice for convenience. (b) Initial state Γ $(K-K)$ exciton. (c) After first phonon scattering K $(K'-K)$ exciton. (d) After second phonon scattering we have a K'-like $(\Gamma-K)$ exciton. (e) e-h interaction brings a $\Gamma-K$ exciton to the K'-exciton $(K-K')$. Open circle denotes empty state (h state can be obtained by time-reversal). (f) Scattered I accounting for $\Gamma \rightarrow K \rightarrow K'$ photon-assisted transitions for $\gamma'/\gamma_o = 0.3$, $\gamma''/\gamma_o = 0.3$, at 0K

with $W_{\boldsymbol{k}'\boldsymbol{k}}$ the transition rate between states with wavevectors $\boldsymbol{k}$ and $\boldsymbol{k}'$, with phonon emission or absorption. For simplicity, we disregard elastic scattering. γ' is the damping rate unrelated to exciton-phonon interaction, $g_{\boldsymbol{k}}$ is the exciton generation rate. The kinetic equation also allows us to account for finite temperature, T, effects[16].

It is convenient to average $f_{\boldsymbol{k}}$ over the in-plane directions of $\boldsymbol{k}$, and consider just the exciton energy distribution function $f(\varepsilon)$. The latter can be recast as:

$$f(\varepsilon) = \sum_{j=-\infty}^{\infty} f_j \delta(\varepsilon_0 - j\hbar\Omega), \tag{17}$$

with f_j satisfying the set of equations:

$$\gamma f_j = \gamma_o \left[f_{j-1}(n_o+1) + f_{j+1} n_o \right] + g_j, \quad j = \ldots, -2, -1, 0, 1, 2, \ldots, \tag{18}$$

where

$$n_o = \frac{1}{\exp\left(\frac{\hbar\Omega}{k_B T}\right) - 1},$$

is the phonon mode occupancy at T, γ_o is the rate of spontaneous phonon emission,

$$\gamma = \gamma_o(2n_o + 1) + \gamma',$$

is the total exciton damping rate. g_j is the exciton generation rate in the state j related to the process of virtual formation of the exciton within the light cone, and its consequent relaxation to the real state with the phonon emission or absorption. Thus, the non-zero values of g_j are:

$$g_1 = g(n_o + 1), \quad g_{-1} = g n_o, \tag{19}$$

with g a parameter. In the main text we replaced the generation rate Eq.(19) with a simplified model with $g_0 = g \neq 0$. This is also valid if elastic scattering is strong and excitons can leave the light cone via static defect scattering.

Since photon emission requires a phonon-induced transition, the intensity of the peak j with phonon-induced energy shift $j\hbar\Omega$ is given by:

$$I_j = f_{j-1}\gamma_o(n_o + 1) + f_{j+1}\gamma_o n_o \propto f_j. \tag{20}$$

The last proportionality is due to the kinetic Eq.(18) and is valid for $j \neq 0, \pm 1$.

Supplementary Note 4. Cascades in a free carriers model

Cascades in phonon-assisted hot PL are possible for free carriers, i.e., for e-h pairs in the continuum states. In this situation the phonon-assisted e/h relaxation is independent, and governed by Eq.(18). Light scattering can be represented as

$$\text{photon} \to \text{electron}, \boldsymbol{k} + \text{hole}, \boldsymbol{k}$$

$$\xrightarrow{\hbar\Omega} \text{electron}, \boldsymbol{k} - \boldsymbol{q} + \text{hole}, \boldsymbol{k} \ldots$$

$$\xrightarrow{\hbar\Omega} \text{electron}, \boldsymbol{k} + \text{hole}, \boldsymbol{k} \to \text{photon}.$$

Accordingly, we expect oscillations in scattered light intensity with period $\hbar\Omega$.

Supplementary Note 5. Intervalley scattering model

We now consider the case of exciton scattering enabled by BZ edge phonons with wavevectors $\boldsymbol{Q} \sim \pm\boldsymbol{K}$. An exciton scatters between Γ valley ($K-K$ or $K'-K'$ exciton) and K/K' valleys ($K'-K$ and $K-K'$ excitons), Supplementary Figure 5a. Photon emission occurs only for states with small wavevectors (∼the photon ones), according to the paths:

$$\text{photon} \to \Gamma \xrightarrow{\hbar\Omega} K \xrightarrow{\hbar\Omega} \text{photon};$$

$$\text{photon} \to \Gamma \xrightarrow{\hbar\Omega} K \xrightarrow{\hbar\Omega} K' \xrightarrow{\hbar\Omega} \text{photon};$$

$$\text{photon} \to \Gamma \xrightarrow{\hbar\Omega} K \xrightarrow{\hbar\Omega} \Gamma \xrightarrow{\hbar\Omega} K \xrightarrow{\hbar\Omega} \text{photon};$$

$$\text{photon} \to \Gamma \xrightarrow{\hbar\Omega} K \xrightarrow{\hbar\Omega} \Gamma \xrightarrow{\hbar\Omega} K' \xrightarrow{\hbar\Omega} \text{photon};\ldots$$

Processes like "$\Gamma \xrightarrow{\hbar\Omega}$ photon" are forbidden due to momentum conservation.

The set of equations describing the processes reads:

$$\gamma_\Gamma f_j^\Gamma = \gamma_o \left[f_{j-1}^K(n_o+1) + f_{j+1}^K n_o\right] + \gamma_o \left[f_{j-1}^{K'}(n_o+1) + f_{j+1}^{K'} n_o\right] + g\delta_{j,0}, \tag{21a}$$

$$\gamma_K f_j^K = \gamma_o \left[f_{j-1}^\Gamma(n_o+1) + f_{j+1}^\Gamma n_o\right] + \gamma_o' \left[f_{j-1}^{K'}(n_o+1) + f_{j+1}^{K'} n_o\right], \tag{21b}$$

$$\gamma_{K'} f_j^{K'} = \gamma_o \left[f_{j-1}^\Gamma(n_o+1) + f_{j+1}^\Gamma n_o\right] + \gamma_o' \left[f_{j-1}^{K}(n_o+1) + f_{j+1}^{K} n_o\right]. \tag{21c}$$

with f_j^Γ, f_j^K, $f_j^{K'}$ the exciton occupancies in the corresponding valley. γ_o and γ_o' are the photon spontaneous emission rates for the scattering processes $\Gamma \leftrightarrow K/K'$ and $K \leftrightarrow K'$, respectively, and the decay rates are:

$$\gamma_\Gamma = \gamma_o(2n_o+1) + \gamma', \quad \gamma_K = \gamma_{K'} = (\gamma_o + \gamma_o')(2n_o+1) + \gamma''.$$

The scattering processes in the e-h picture are presented in Supplementary Figure 5b-e. These demonstrate that γ_o and γ_o' are different, because the second process ($K \leftrightarrow K'$) needs additional Coulomb interaction, Supplementary Figure 5c-e. Thus, direct phonon-induced $K \leftrightarrow K'$ transfer is impossible, and the corresponding process takes place via an intermediate state with e in the Γ valley. The results of the calculations in Supplementary Figure 5f demonstrate that pronounced I oscillations should take place at low T (below the phonon energy). These oscillations are not observed experimentally in Figure 2b of the main

text (T=78K), ruling-out this pathway.

---

[1] Ivchenko, E., Lang, I. & Pavlov, S. Theory of resonance secondary radiation emitted by semiconductors. *Phys. Solid State* **19**, 1610 (1977).

[2] Goltsev, A., Lang, I., Pavlov, S. & Bryzhina, M. Multiphonon resonance raman scattering and spatial distribution of electrons and holes. *Journal of Physics C: Solid State Physics* **16**, 4221 (1983).

[3] Belitsky, V. *et al.* Outgoing excitonic resonance in multiphonon raman scattering from polar semiconductors. *Physical Review B* **52**, 11920 (1995).

[4] Wang, G. *et al.* Colloquium: Excitons in atomically thin transition metal dichalcogenides. *Reviews of Modern Physics* **90**, 021001 (2018).

[5] Zeyher, R. Theory of multiphonon raman spectra above the energy gap in semiconductors. *Solid State Communications* **16**, 49–52 (1975).

[6] He, M. *et al.* Valley phonons and exciton complexes in a monolayer semiconductor. *Nature communications* **11**, 618 (2020).

[7] Jin, Z., Li, X., Mullen, J. T. & Kim, K. W. Intrinsic transport properties of electrons and holes in monolayer transition-metal dichalcogenides. *Phys. Rev. B* **90**, 045422 (2014).

[8] Danovich, M., Aleiner, I. L., Drummond, N. D. & Fal'ko, V. I. Fast relaxation of photo-excited carriers in 2-d transition metal dichalcogenides. *IEEE Journal of Selected Topics in Quantum Electronics* **23**, 168–172 (2016).

[9] Glazov, M. *et al.* Intervalley polaron in atomically thin transition metal dichalcogenides. *Physical Review B* **100**, 041301 (2019).

[10] Christiansen, D. *et al.* Phonon sidebands in monolayer transition metal dichalcogenides. *Physical review letters* **119**, 187402 (2017).

[11] Shree, S. *et al.* Observation of exciton-phonon coupling in mose 2 monolayers. *Physical Review B* **98**, 035302 (2018).

[12] Martin, E. W. *et al.* Encapsulation narrows excitonic homogeneous linewidth of exfoliated mose $_2$ monolayer. *arXiv preprint arXiv:1810.09834* (2018).

[13] Schneider, C., Glazov, M. M., Korn, T., Höfling, S. & Urbaszek, B. Two-dimensional semiconductors in the regime of strong light-matter coupling. *Nature Comms.* **3**, 2695 (2018).

[14] Ivchenko, E., Pikus, G., Razbirin, B. & Starukhin, A. Optical orientation and alignment of free excitons in gase during resonant excitation-theory. *ZhETF* **72**, 2230–2245 (1977).

[15] Glazov, M. Quantum interference effect on exciton transport in monolayer semiconductors. *Physical Review Letters* **124**, 166802 (2020).

[16] Tserkovnikov, Y. A. Kinetic equations in the method of two-time finite-temperature green's functions. i. renormalization of the collision integral. *Theoretical and Mathematical Physics* **96**, 1013–1026 (1993).
